# Matrix metalloproteinase-3 and the 7-joint ultrasound score in the assessment of disease activity and therapeutic efficacy in patients with moderate to severe rheumatoid arthritis

**DOI:** 10.1186/s13075-017-1449-z

**Published:** 2017-11-15

**Authors:** Ling Zhou, Geng Wang, Xin Liu, Jing Song, Ling Chen, Huji Xu

**Affiliations:** Department of Rheumatology and Immunology, Changzheng Hospital, Naval Medical University, 415 Fengyang Road, Shanghai, 200003 China

**Keywords:** Rheumatoid arthritis, Matrix metalloproteinase-3, Articular ultrasonography

## Abstract

**Background:**

This study aimed to investigate the reliability and validity of serum matrix metalloproteinase-3 (MMP-3) levels and articular ultrasound (US) scores in assessing disease activity and therapeutic response in rheumatoid arthritis (RA) patients.

**Methods:**

A total of 151 RA patients were enrolled, of whom 22 were treated with certolizumab pegol (Cimzia, CZP). The RA patients were divided into the following four subgroups according to their disease activity score in 28 joints (DAS28): stable, mild activity, moderate activity, and high activity. Forty-three healthy controls were simultaneously studied. The serum MMP-3 levels and 7-joint US (US7) scores of all subjects were determined. The patients who were treated with CZP were subsequently followed for 6 months.

**Results:**

The serum MMP-3 levels of all the RA patients were significantly higher than those of healthy controls, and those of patients with moderate and severe RA were significantly higher than those of patients with stable RA. The US7 scores of patients with severe RA were significantly higher than those of patients in other groups. Using the DAS28 as a reference standard, the corresponding cutoff value of MMP-3 was 70.5 ng/ml. After CZP treatment, the MMP-3 levels and US7 scores were significantly decreased at week 2, and the mean changes in US7 scores at weeks 12 and 24 were significantly higher in both groups with American College of Rheumatology 50% positive response (ACR50) and ACR 70% positive response (ACR70) than in the negative groups.

**Conclusion:**

Serum MMP-3 and the US7 scores could both effectively reflect disease activity and therapeutic responses in patients with moderate to severe RA.

**Trial registration:**

CTR20140405 (RA0044), CTR20140405: A phase 3, Multicenter, Double-blind, Placebo Controlled, Parallel Group, Randomized, 24-Week Study to Evaluate the Safety and Efficacy of Certolizumab Pegol as Additional Medication to Methotrexate in Chinese Subjects With Active Rheumatoid Arthritis Who Have an Incomplete Response to Methotrexate, Registered on 13 June 2014.

CTR20140412 (RA0078), CTR20140412: A phase 3, Multicenter, Open-label Extension Study to Assess the Safety and Efficacy of Certolizumab Pegol as Additional Medication to Methotrexate in Chinese Subjects With Active Rheumatoid Arthritis Who Participated in RA0044, Registered on 02 July 2014.

## Background

Rheumatoid arthritis (RA) is a chronic, serious systemic autoimmune disease that is primarily characterized by multi-joint synovitis [1]. Therefore, comprehensive approaches to assessing joint damage in patients with RA are pivotal for the early diagnosis and treatment of RA in clinical settings [2]. Currently, approaches to the clinical assessment of RA disease activity, such as the disease activity score in 28 joints (DAS28) require complicated algorithms with multiple parameters, including the number of swollen and tender joints, erythrocyte sedimentation rate (ESR), C-reactive protein (CRP) levels, and visual analogue scale (VAS) score, etc. Moreover, the 28-joint ultrasound (US28) score and the simplified disease activity index (SDAI) and clinical disease activity index (CDAI) considers 28 joints, which is simple but could be time-consuming. Even so, these parameters can only indirectly reflect damage to cartilage and bone in RA. Thus, accurate and simple methods are urgently needed to assess disease activity in patients with RA.

Matrix metalloproteinases (MMPs), which contain the Zn2+ ion, are the most important proteases participating in extracellular matrix degradation [3]. In recent years, it has been reported that inflammatory cytokines can induce the secretion of MMP-1 and MMP-3, which are important factors in the degradation of cartilage and bone matrix [4]. Houseman et al. found that MMP-3 and anti-cyclic citrullinated peptide (anti-CCP) antibodies were strongly predictive of joint damage in patients with RA [5]. In addition, it was previously reported that MMP-3 levels in the synovium of patients with RA were significantly elevated; moreover, the gene that encodes MMP-3 was found to be overexpressed in the synovium [6]. These data suggest that MMP-3 is a biomarker of cartilage degeneration. However, few studies have reported that MMP-3 is an early predictor of disease improvement and therapeutic response.

Articular ultrasound has become an important diagnostic technique for RA owing to its versatile nature of being non-invasive, non-radiation, economic, portable, and providing real-time dynamic monitoring, etc. Articular ultrasound is superior to traditional radiography and clinical examination and is equivalent to computed tomography (CT) and magnetic resonance imaging (MRI) [7]. Gray-scale ultrasound can be used to observe fluid, the synovium, tendons, periarticular soft tissue, cartilage, and bone erosion dynamically in real time. A previous study suggested that power Doppler ultrasound (PDUS) is a more sensitive method for revealing disease activity than other clinical assessments [8]. At present, there is no standard for accurate evaluation of RA using ultrasound. Currently, the most simple and convenient clinical system is the 7-joint ultrasound (US7) score, which was proposed by Backhau et al. [9]. The US7 scoring system reflects the evaluation of soft tissue lesions and bone destructive changes in a set of joints. Hammer et al. analyzed the value of evaluating different numbers of joints using the US7, US12, US28, US44, and US78 in evaluating RA, finding that the US7 and US78 scores had similar sensitivity [10].

MMP-3 is a potential serum marker of cartilage degradation in RA and is related to disease activity. However, MMP-3 has rarely been used in the classification of RA activity. Ultrasound is a simple and practical approach that can directly reflect the progress of patients with RA as identified on clinical imaging. Therefore, this study attempts, for the first time, to combine the two approaches in the evaluation of disease activity and treatment efficacy in different categories of severity of RA. The ultimate aim of this work is to identify a more accurate and simpler method of monitoring disease activity and estimating treatment efficacy according to disease severity to guide clinical decisions in patients with RA.

## Methods

### Study subjects

A total of 151 patients with RA were recruited from the Department of Rheumatology and Immunology of Shanghai Changzheng Hospital (Shanghai, China) from December 2014 to June 2015. All study subjects with RA met the 1987 American College of Rheumatology (ACR) criteria for RA.

Clinical information was recorded for all patients, including disease duration, the number of joints with tenderness upon touching and swelling of the joints. According to the DAS28, a component of the European League Against Rheumatism response criteria, the patients were separated into four subgroups: stable or remissive, mild (2.6 ≤ DAS28 < 3.2), moderate (3.2 ≤ DAS28 < 5.1), and highly active (DAS28 ≥ 5.1). Of the 151 patients with RA, 22 had received certolizumab-pegol (Cimzia, CZP) and methotrexate treatment, with the following dosing parameters: biweekly intravenous injections of 400 mg CZP for the first 6 weeks and 200 mg biweekly for the subsequent 18 weeks, in combination with methotrexate (10 mg once per week during the course of treatment). This has been proven to effectively treat moderate to severe RA [11]. Prednisone was discontinued at least 1 month before the study start.

In addition, 43 healthy subjects with no immune system diseases, liver or kidney diseases, infections, or cancers were included as controls from the Medical Examination Center of Shanghai Changzheng Hospital.

All the recruited subjects who participated in the study had provided informed consent. The study protocol has been approved by the Medical Ethics Committee of Shanghai Changzheng Hospital.

### Specimen collection

Serum was collected from centrifuged venous blood (5 ml) from patients and healthy volunteers in the fasting state. The specimens were centrifuged within 2 hours and preserved at − 20 °C for subsequent testing.

### Determination of serum levels of anti-CCP antibodies, MMP-3, and other markers

The level of anti-CCP antibodies was determined using an enzyme-linked immunosorbent assay, and MMP-3 levels were tested by immunoturbidimetry, following the assay kit instructions (Shanghai Huachen Reagent Co., Shanghai, China). The examination was conducted using automatic biochemical analyzers. The ESR was detected using the Westergren method (mm/h), and the level of CRP was detected using automatic immune rate nephelometry (mg/L).

### Ultrasound assessment

Ultrasound assessments of seven articular regions were conducted by two experienced sonographers using a Philips HD9 color Doppler ultrasound device equipped with a high-frequency linear array probe (5–12 MHz) in a double-blind fashion. These assessments were performed within 4 hours of the clinical tests. The procedure standards were in accordance with the guidelines for musculoskeletal ultrasonography, which were jointly developed by the American Institute of Ultrasound Medicine (AIUM) and American College of Radiology. The seven articular regions were the dominant wrist, the second and third metacarpophalangeal (MCP2 and MCP3) and proximal interphalangeal (PIP2 and PIP3) joints, and the second and fifth metatarsophalangeal (MTP2 and MTP5) joints. Three characteristics of each joint are assessed, namely, synovitis, tenosynovitis, and bone erosion. Synovitis was rated from 0 to 3, corresponding to none, mild, moderate, and severe. Tenosynovitis and bone erosion were classified as positive or negative and scored 1 or 0, accordingly. If synovitis was detected, additional observations were made of the blood flow signal using color Doppler [12]. The pulse repetition frequency (PRF) was tuned to the minimum frequency range (0.7–1.0 kHz). The Doppler signal was classified into the three following categories: (1) a few spots of the blood flow signal; (2) a continuous flow signal with an area <50% of the joint cavity; and (3) blood flow in more than 50% of the joint cavity. The synovitis, tenosynovitis, bone erosion scores, and respective flow signals were recorded for seven joints, with a maximum score of 94.

### Statistical analysis

All statistical analyses were performed with GraphPad Prism 6.0c software (GraphPad Software, Inc., La Jolla, CA, USA). Normally distributed data are presented as the mean ± standard deviation $\left(\overline{\mathrm{x}}\pm \mathrm{S}\right)$, and non-normally distributed data are presented as the mean (P25, P75). The Wilcoxon test was used for inter-group comparisons. The validity of MMP-3, US7 and their combination (MMP-3 + US7) in classifying the disease activity of RA were evaluated using receiver operating characteristic (ROC) curve analysis. The relationship between the various clinical and laboratory parameters was tested using Spearman correlation analysis. A two-sided *p* value less than 0.05 was considered statistically significant.

## Results

### General characteristics of participants

There were 151 patients with RA, including 135 women and 16 men, with ages from 21 to 72 years (mean 47.82 ± 13.15 years). The healthy controls were 23 women and 20 men, with ages from 24 to 66 years (mean 40.28 ± 19.12 years) (Table 1). Of the 22 patients with RA receiving CZP treatment, there were 19 women and 3 men, with ages from 25 to 57 years (mean 44 ± 10.64 years).

**Table 1:** Demographic characteristics and the selected laboratory values for 151 patients with RA

	Stable RA	Mild RA	Moderate RA	Severe RA	RA	Control
	33 patients	32 patients	35 patients	51 patients	151 patients	43 patients
Age^a^	48.23 ± 10.28	44.12 ± 12.01	45.18 ± 11.01	49.14 ± 12.51	47.82 ± 13.15	40.28 ± 19.12
Sex (male/female)	5/28	4/28	4/30	3/49	16/135	20/23
Disease duration [20]^b^	6 (3 ~ 10)	5 (2 ~ 10)	3 (0 ~ 5)	10 (1 ~ 11)**	7(1 ~ 11)	
Number of joints with tenderness^b^	1 (0 ~ 10)	2 (0 ~ 10)	3 (0 ~ 9)	8 (6 ~ 14)***	5(2 ~ 13)	
Number of joints with swelling^b^	0 (0 ~ 4)	1 (0 ~ 4)	3 (0 ~ 4)**	3 (0 ~ 7)	2 (0 ~ 6)	
DAS28^b^	2.13 (1.83 ~ 2.32)	2.98 (2.81 ~ 3.04)**	4.12 (3.61 ~ 4.58)**	6.21 (5.44 ~ 6.71)***	3.82 (2.81 ~ 5.635)	
ESR^b^	18.04 (10.21 ~ 40.27)	27.05 (14.25 ~ 45.75)**	39.10 (23.21 ~ 51.52)**	45.22 (33.53 ~ 58.51)***	37.00 (21.50 ~ 52.00)	
HAQ^b^	5.05 (2.21 ~ 10.32)	7.04 (4.25 ~ 19.98)**	12.05 (6.03 ~ 19.50)**	20.03 (12.04 ~ 27.12)***	12.04 (5.40 ~ 20.12)	
Anti-CCP^b^	185.04 (33.20 ~ 678.41)	209.90 (81.62 ~ 957.71)	195.71 (38.27 ~ 178.12)	192.70 (16.48 ~ 294.12)	108.7 (34.7 ~ 607.3)	
US7^b^	0.8 (0.3 ~ 2.4)	2.2 (0.8 ~ 5.5)	3.2 (0.9 ~ 6.2)	7.8 (2.6 ~ 11.2) ***	4.0 (0.5 ~ 9.7)	
MMP3^b^	33.40 (22.60 ~ 678.42)	54.25 (33.23 ~ 81.60)	105.10 (61.70 ~ 172.71)^***^***	363.11 (161.52 ~ 475.92)^***^***	99.3 (23.65 ~ 415.83) ^***^	35.20 (25.90 ~ 48.90)

*RA* rheumatoid arthritis, *CCP* cyclic citrullinated peptide, *CRP* C-reactive protein, *DAS28* disease activity score in 28 joints, *ESR* erythrocyte sedimentation rate, *HAQ* health assessment questionnaire, *MMP*-*3* matrix metalloproteinase-3, *US7* the 7-joint ultrasound score

^a^The data are presented as the mean ± standard deviation.

^b^The data are presented as the median and interquartile range

Compared with patients with stable RA, ***p < 0.001, **p < 0.01, *p < 0.05; compared with the healthy controls, ^***^ *p* < 0.001, ^**^ *p* < 0.01 and ^*^ *p* < 0.05.

### Serum MMP-3 levels, articular US7 scores, and other indexes of patients with RA and controls

The ESR, DAS28, and HAQ in each group of patients with active RA were significantly higher than those in patients with stable RA (*p* < 0.01 or *p* < 0.001). There was no significant difference in the levels of anti-CCP antibodies between the analyzed groups (*p* > 0.05) (Table 1).

**Fig. 1:**
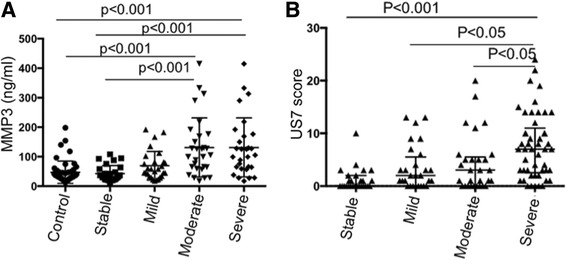
Serum matrix metalloproteinase-3 (MMP-3) levels (**a**) and 7-joint ultrasound (US7) scores (**b**) in the different groups of patients with active rheumatoid arthritis (RA). **a** MMP-3 in patients with RA with moderate and severe disease activity were significantly higher than in patients with stable RA (105.10 vs 33.40, *p* < 0.001; 363.11 vs 33.40, *p* < 0.001). No significant differences were found between the group with mild RA and the group with stable RA or the normal control group (54.25 vs 33.40, *p* > 0.05; 54.25 vs 35.20, *p* > 0.05). **b** The US7 scores in patients with severe active RA were significantly higher than those in patients in the stable, mild, and moderate RA groups (7.8 vs 0.8 *p* < 0.001; 7.8 vs 2.2 *p* < 0.05; 7.8 vs 3.2 *p* < 0.05). No significant differences were found between patients with mild or moderate RA activity and those with stable activity (*p* > 0.05)

The serum MMP-3 levels in all patients with RA were significantly higher than those in the healthy controls (*p* < 0.001). The MMP-3 levels in patients with moderate and severe RA were both significantly higher than those in patients with stable RA (all *p* < 0.001). However, there was no significant difference in the levels of this marker between mild patients with RA and those with stable RA or controls (*p* > 0.05, Fig. 1a and Table 1).

The US7 scores in patients with severe active RA were significantly higher than those in patients with stable, mild, and moderate RA (*p* < 0.001, *p* < 0.05 and *p* < 0.05, respectively). However, no significant differences in the US7 score were observed between the mild or moderate RA group and the stable RA group (Fig. 1b).

### Correlations between MMP-3 and other biomarkers

**Fig. 2:**
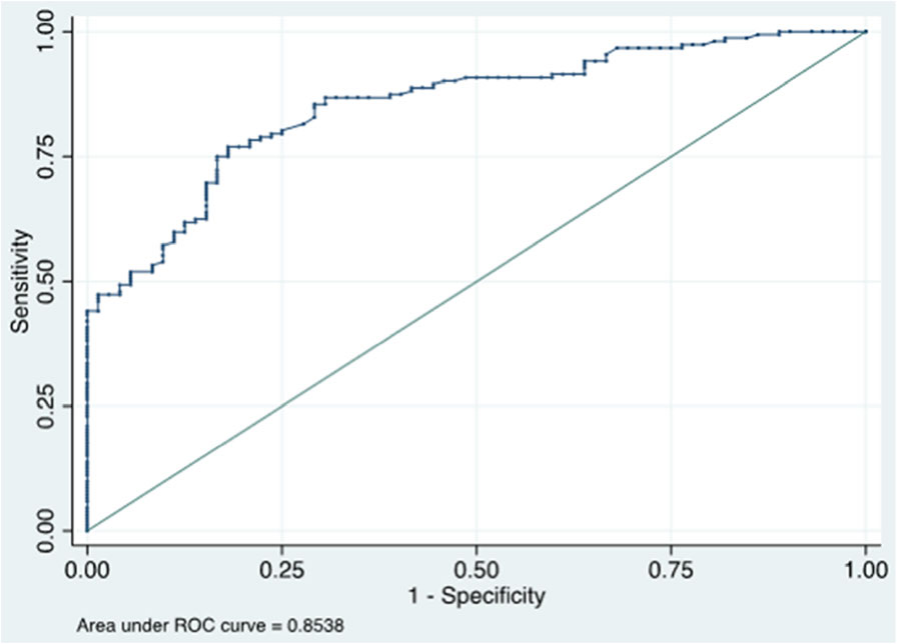
Receiver operating characteristic (ROC) curve of matrix metalloproteinase-3 (MMP-3) for classification of disease activity in rheumatoid arthritis (RA). Setting the disease activity score in 28 joints (DAS28) as the reference standard, the sensitivity of MMP-3 in classifying the disease activity of RA was determined using a ROC curve. The corresponding cutoff value in the ROC curve of MMP-3 is 70.5 ng/ml (area under the curve 0.8538, *p* < 0.0001)

The US7 score was positively correlated with MMP-3 in patients with RA (*r* = 0.566, *p* < 0.001, Table 2). Using the DAS28 as the reference standard, the corresponding cutoff value in the ROC curve for MMP-3 was 70.5 ng/ml (area under the curve = 0.8538, *p* < 0.0001, Fig. 2). The sensitivity and specificity of US7 combined with MMP-3 were 58.8% and 92.6%, respectively. The difference in the US7 scores between MMP-3-negative and MMP-3-positive patients was significant (1.463 ± 0.3085 vs 6.457 ± 0.5295, *p* < 0.0001, Fig. 2).

**Table 2:** Correlations between MMP-3 or US7 scores and other variables

	US7	CCP	ESR	DAS28	HAQ	Synovitis	Tenosynovitis	Bone erosion
MMP-3	0.586***	−0.239**	0.576***	0.513***	0.471***	0.525***	0.348***	0.339**
US7		−0.174	0.384***	0.487***	0.434***	0.959***	0.492***	0.456***
Anti-CCP			−0.069	−0.216*	−0.106	−0.160	−0.126	−0.188
ESR				0.495***	0.310**	0.376***	0.238	0.246
DAS28					0.546***	0.445***	0.312*	0.282
HAQ						0.408***	0.277*	0.157
Synovitis							0.394**	0.278
Tenosynovitis								0.363**

*CCP* cyclic citrullinated peptide, *CRP* C-reactive protein, *DAS28* disease activity score in 28 joints, *ESR* erythrocyte sedimentation rate, *HAQ* health assessment questionnaire, *MMP*-*3* matrix metalloproteinase-3, *US7* 7-joint ultrasound score

***p < 0.001,**p < 0.01,*p < 0.05

### MMP-3 and US7 in evaluating the efficacy of CZP treatment

**Fig. 3:**
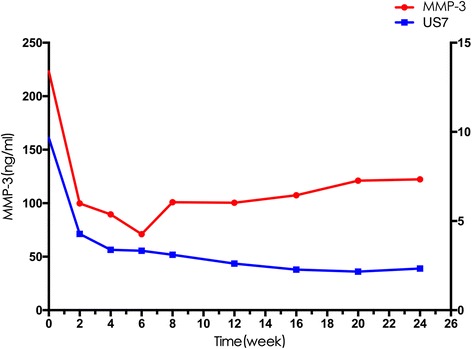
Matrix metalloproteinase-3 (MMP-3) and 7-joint ultrasound (US7) scores in 22 patients with rheumatoid arthritis (RA) after treatment with certolizumab pegol (CZP). US7 scores were compared between the MMP-3-positive and the MMP-3-negative groups. Patients’ MMP-3 levels and US7 scores were significantly decreased at week 2 compared with baseline levels

The MMP-3 levels and US7 scores in the 22 patients with RA who were treated with CZP were significantly decreased at week 2 compared with baseline levels (*p* < 0.001 and *p* < 0.01, respectively; Table 3 and Fig. 3).

**Table 3:** MMP-3, US7 scores, and clinical characteristics of 22 patients with RA being treated with CZP

	Weeks (W) after treatment			
	W0	W2	W12	W24
DAS28	6.17 ± 0.86	4.365 ± 1.12^***^	3.62 ± 1.32^***^	3.43 ± 1.26^***^
US7	8.7 ± 8.61	3.1 ± 3.09^**^	2.35 ± 2.85^**^	2.1 ± 2.51^**^
MMP-3	214.8 ± 160.4	94.11 ± 74.96^***^	98.86 ± 114.7^***^	122.7 ± 167.7^*^
CRP	12.40 ± 15.01	6.05 ± 7.338	5.45 ± 8.27^**^	6.5 ± 15.22
Anti-CCP	363.0 ± 385.6	386.7 ± 454.2	339 ± 374.2	300.3 ± 323.7
HAQ	22.2 ± 14.7	13.85 ± 10.64^***^	12.5 ± 9.95^**^	12.9 ± 10.7^***^
ESR	60.35 ± 22.78	38.95 ± 16.96^***^	29.75 ± 14.59^***^	37.0 ± 21.8^***^
Synovitis	3.70 ± 3.511	2.35 ± 2.98^*^	2.25 ± 2.845^*^	1.3 ± 2.658^**^
Synovial blood flow	1.85 ± 2.368	0.85 ± 1.63^*^	0.65 ± 1.424^*^	0.4 ± 0.821^**^
Tenosynovitis	0.25 ± 0.71	0.05 ± 0.22	0.05 ± 0.23	0
Tenosynovial blood flow	0.25 ± 0.71	0	0	0
Bone erosion	0.65 ± 1.13	0.5 ± 1.0	0.45 ± 0.99	0.3 ± 0.57

*RA* rheumatoid arthritis, *CZP* certolizumab pegol, *CCP* cyclic citrullinated peptide, *CRP* C-reactive protein, *DAS28* disease activity score in 28 joints, *ESR* erythrocyte sedimentation rate, *HAQ* health assessment questionnaire, *MMP*-*3* matrix metalloproteinase-3, *US7* 7-joint ultrasound score

Compared with the baseline level (W0), ***p < 0.001, **p < 0.01, *p < 0.05

The mean changes from baseline in the US7 scores (ΔUS7) at both week 12 and week 24 were significantly greater in the ACR 50% response (ACR50) and ACR70 groups (i.e., ACR50-positive and ACR70-positive) than in patients who did not achieve these responses (ACR50-negative and ACR70-negative) (all *p* < 0.001, Table 4). The mean changes in ΔMMP-3 from baseline were significantly greater in both the ACR50-positive and ACR70-positive groups than those in the negative response groups at week 24 (all *p* < 0.01, Table 4).

**Table 4:** The mean changes in clinical indicators from baseline at weeks 12 and 24 in both ACR50 and ACR70 negative groups and ACR50 and ACR70 positive groups

		ACR50		ACR70	
		Negative	Positive	Negative	Positive
ΔDAS28	W12	2.304 ± 1.626	2.688 ± 0.996	2.583 ± 1.257	2.51 ± 1.255
	W24	2.536 ± 1.598	2.857 ± 0.962	2.591 ± 1.314	2.898 ± 1.099
ΔMMP-3	W12	84.914 ± 32.938	122.599 ± 149.654	102.92 ± 66.121	133.385 ± 182.232
	W24	69.139 ± 33.066	124.365 ± 107.828^**^	76.244 ± 49.253	127.828 ± 220.685^**^
ΔUS7	W12	1.143 ± 1.215	9.154 ± 8.061^***^	1.75 ± 1.658	13.25 ± 7.704^***^
	W24	0.857 ± 1.676	9.692 ± 7.836^***^	1.5 ± 2.224	11.7 ± 7.775^***^
ΔCRP	W12	3.571 ± 4.315	8.769 ± 10.109	6.250 ± 7.689	8.000 ± 10.664
	W24	3.00 ± 5.888	7.385 ± 11.507	5.600 ± 9.582	6.100 ± 16.032
ΔAnti-CCP	W12	68.01 ± 166.238	69.32 ± 149.09	58.1 ± 143.488	72.145 ± 108.321
	W24	75.47 ± 158.87	77.35 ± 127.76	69.68 ± 122.58	77.65 ± 148.53
ΔHAQ	W12	8.429 ± 8.904	10.385 ± 13.556	7.33 ± 8.283	13.250 ± 15.926
	W24	9.429 ± 9.502	9.231 ± 8.757	8.0 ± 9.534	10.6 ± 8.235
ΔESR	W12	28.714 ± 22.134	31.615 ± 21.964	35.41 ± 20.291	32.75 ± 21.446
	W24	25.429 ± 19.68	22.231 ± 28.94	27.8 ± 20.065	18.90 ± 30.552

The Δ symbol represents changes from baseline

*ACR50* American College of Rheumatology 50% response, *ACR70* ACR 70% response, *CCP* cyclic citrullinated peptide, *CRP* C-reactive protein, *DAS28* disease activity score in 28 joints, *ESR* erythrocyte sedimentation rate, *HAQ* health assessment questionnaire, *MMP*-*3* matrix metalloproteinase-3, *US7* 7-joint ultrasound score

Compared with the negative group, ***p < 0.001, **p < 0.01, *p < 0.05

## Discussion

RA is a highly prevalent chronic inflammatory disease. Accurate and simple methods are urgently needed to assess both RA disease activity and treatment efficacy in patients with RA. This study was performed to determine the role of MMP-3 measurements and the US7 score in the assessment of RA activity. The results indicate the effectiveness of the combined evaluation of serum MMP-3 and the US7 score in the assessment of RA activity and therapeutic efficacy in patients with RA.

The current study found that MMP-3 was significantly increased in patients with RA, especially among patients with moderate to severe disease activity. Moreover, the US7 scores in severe active RA were significantly higher than in stable, mild, and moderate RA. These aroused our interest in whether MMP-3 was associated with US7 scores in the evaluation of RA. The results of Spearman correlation analysis indicated that MMP-3 and US7 are strongly correlated (*r* = 0.586, *p* < 0.001). This result is consistent with the study of Gora et al., who reported correlation between US28 and MMP-3 [13]. Therefore, MMP-3 and US7 were good assessment biomarkers of moderate to severe RA. We also found that correlation between MMP-3 and the US7 synovitis score was much stronger than for tendon sheath synovitis and bone destruction scores. These data are supported by clinical observations that MMP-3 is highly suggestive of joint inflammation in clinical practice and the level of it was elevated both in the serum and the synovial fluid of patients with RA and other forms of arthritis [14, 15]. This may be attributed to the correlation between serum MMP-3 and inflammatory biomarkers and imaging changes [16, 17], while patients with RA with mild or stable disease and minimal synovial inflammation are usually free from massive cartilage and bone destruction [18]. In addition, ROC curve analysis identified a 70.5 ng/ml cutoff value for MMP-3 for diagnosis of severe RA, and this performed well in detecting joint involvement (area under the curve 0.8538, *p* < 0.0001). Using cutoff values as the classification criteria, we found that the US7 scores in the MMP-3-positive group were much higher than those in the MMP-3-negative group. Thus, serum MMP-3 could be used as a pre-evaluation standard to assist or reduce the burden on medical staff.

In addition, to verify the effect of combined evaluation of MMP-3 and US7 in patients with moderate to severe RA treated with methotrexate and CZP, the disease activity and outcome measure in the clinical follow up of patients with RA was assessed. Patients’ MMP-3 levels and US7 scores decreased significantly from baseline in the second week. In addition, the downward trends in MMP-3 and US7 scores were correlated, suggesting that MMP-3 and articular ultrasound can sensitively and stably detect a therapeutic response in clinical practice. This result further suggests that both markers can be used as indices of efficacy. Moreover, no significant ultrasound-detected changes were associated with minor joint inflammation in patients with stable, mild activity. However, positive US7 scores were observed in the patients with stable and mild RA, suggesting that clinically stable patients still suffer from synovial hyperplasia and bone damage. This result is consistent with a recent study by Nguyen et al., who suggest that ultrasound can detect even residual synovitis in patients with RA in clinical remission, a finding that was predictive of RA recurrence and progression [19].

There were some limitations in this study: the follow-up sample size was quite small, rendering it difficult to stratify the sample into groups. In addition, the frequency of the ultrasonic probe, which was 5–12 MHz, and the fact that the US7 score is based on only seven articular areas, means the large joints were excluded. The US7 score can also be influenced by subjective factors. Hence, to verify the role of MMP-3 measurement and the US7 score in assessing disease activity in moderate to severe RA, it will be imperative to repeat these analyses with a higher-frequency probe and to expand the sample size.

## Conclusions

In conclusion, this study confirmed that serum levels of MMP-3 and the US7 score are strongly correlated and can effectively reflect disease activity in patients with moderate to severe RA. After treatment with CZP, MMP-3 and the US7 score were able to sensitively and stably detect therapeutic responses in patients with moderate to severe RA. Therefore, the combination of serum MMP-3 measurements and the US7 score could be a simple and practical assessment approach for detection of RA disease activity and therapeutic efficacy in the clinical setting. Moreover, the MMP-3 value 70.5 ng/ml may be considered a pre-assessment indicator of severe RA.

## Abbreviations

ACR: American College Rheumatology
AIUM: American Institute of Ultrasound Medicine
Anti-CCP: Anti-cyclic citrullinated peptide
CRP: C-reactive protein
CZP: Certolizumab pegol
DAS28: Disease activity score in 28 joints
ESR: Erythrocyte sedimentation rate
HAQ: Health assessment questionnaire
MCP: Metacarpophalangeal joints
MMP: Matrix metalloproteinase
PDUS: power Doppler ultrasound
PIP: Proximal interphalangeal joints
PRF: Pulse repetition frequency
RA: Rheumatoid arthritis
ROC: Receiver operating characteristic
US7: The 7-joint ultrasound score
VAS: Visual analogue scale
